# Real-World Use and Outcomes of Hard-To-Heal Wounds Managed With Porcine Placental Extracellular Matrix

**DOI:** 10.7759/cureus.76262

**Published:** 2024-12-23

**Authors:** Caroline Fife, Ben LeBoutillier, Cristin Taylor, Brad T Marcinek

**Affiliations:** 1 Medicine, Intellicure, LLC, The Woodlands, USA; 2 Data Management, Intellicure, LLC, The Woodlands, USA; 3 Medical Affairs, Convatec, Bridgewater, USA

**Keywords:** cellular acellular and matrix-like product, hard-to-heal wounds, limb- or life-threatening, porcine placental extracellular matrix, real-world data, us wound registry

## Abstract

Real-world data are a highly valuable resource in determining the efficacy of novel products in challenging populations, especially in wound care. This study retrospectively analyzed the real-world performance of porcine placental extracellular matrix (PPECM; InnovaMatrix® AC, Convatec Triad Life Sciences, LLC, Memphis, TN, USA), a novel cellular, acellular, and matrix-like product for the management of hard-to-heal wounds. The US Wound Registry (USWR), which comprises aggregated and structured electronic health records from 502 wound practices, provided a deidentified dataset collected from October 10, 2022 to March 25, 2024, containing 76,278 patients (248,278 wounds). Screening for PPECM usage identified 60 wounds in 41 patients. The median age was 74 (IQR: 66-80) years; 20 (49%) had impaired ambulation, five (12%) autoimmune diseases, and five (12%) peripheral arterial diseases. The most common wounds included 18 (30%) chronic ulcers, 12 (20%) diabetic ulcers, seven (12%) pressure ulcers or injuries, seven (12%) dehisced surgical wounds, and six (10%) venous leg ulcers. Median surface area was 1.50 (IQR: 0.42-4.69) cm²; 31 (52%) of the wounds were limb/life-threatening (L/LT); nine (17%) were present >1 year; 52 (87%) had bioburden/infection. Only two (3%) of the wounds had no necrotic tissue at the initial application. Following PPECM management, 32 (53%) of the wounds closed (of which 14 (44%) were L/LT) and five (8%) had major improvement (three (60%) L/LT). No adverse events were reported. This is the first clinical study of PPECM and demonstrates the real-world safety and efficacy of PPECM in the management of hard-to-heal wounds in a complex population.

## Introduction

Hard-to-heal wounds are increasingly prevalent, affecting 16% of Medicare beneficiaries, according to a 2019 analysis [1]. Despite the growing need for effective treatments, clinical studies evaluating novel interventions have been hindered by substantial design flaws. Such studies are limited by their focus on wounds that poorly represent the full spectrum of those encountered in clinical practice [2]. Most prospective trials in wound care focus on diabetic foot ulcers (DFUs) and venous leg ulcers (VLUs) [3]. However, analyses of the Medicare population [1,4] and patients at wound care centers [5] indicate that DFUs and VLUs account for less than half of identified hard-to-heal wound etiologies. In fact, the hard-to-heal wound is a heterogeneous category that also encompasses traumatic wounds, pressure ulcers/injuries, arterial ulcers, wounds resulting from surgical complications, dermatological conditions, and otherwise unclassified ulcers [1]. Prospective studies enroll relatively healthy subjects with acute, superficial, and noninfected wounds [2,3,6]. Acute wounds typically heal within four to 12 weeks, whereas chronic, hard-to-heal wounds can remain open for months or even years [7,8]. Hard-to-heal longer-duration wounds are usually excluded from prospective studies, as are patients with autoimmune diseases, renal failure, ischemia, and limb/life-threatening (L/LT) wounds [2].

For over 15 years, researchers have acknowledged that these limitations have diminished the value of randomized controlled trials (RCTs) conducted in wound care, which demonstrate efficacy under idealized conditions that exclude many real-world patients [2,3]. The disparities between RCT and real-world populations were established by Serena et al., who compared the demographics and clinical characteristics of RCT participants with those of real-world patients within a consortium of wound care clinics. The VLUs of real-world patients were five times larger than those of subjects enrolled in RCTs, and 44% of real-world DFUs were Wagner Grades 3-5, whereas only Wagner Grade 1 and 2 DFUs were enrolled in RCTs [2].

In response to the limitations of prospective studies in wound care, there has been an increasing drive to accrue real-world data (RWD), which are expected to provide more generalizable evidence about the clinical effectiveness of treatments among typical patients and generate key insights to inform healthcare decision-making [9-11]. RWD is collected from medical/administrative records, registries, surveys, and digital health technologies, which present a broad range of experiences related to patient health and care delivery. Such data can be of value in assessing the performance of novel wound products in routine clinical practice.

Cellular, acellular, and matrix-like products (CAMPs) are a class of novel treatments for hard-to-heal wounds and include therapeutic extracellular matrix (ECM) products that act as 3D scaffolds to support the natural healing process in hard-to-heal wounds [12-14]. When CAMPs are applied to wounds, including wounds that have stalled, they have the ability to further support the ideal environment required for wound closure [15]. Results have shown that when used as an adjunct to standard of care (SOC), CAMPs lead to an increase in the number of ulcer-free months and increase the probability of healing [16,17]. Medicare patients who have received advanced CAMP treatment, compared to those who received no advanced treatment, had the best outcomes [18]. Furthermore, multiple analyses have demonstrated the cost-effectiveness of CAMPs, specifically shortened treatment times, improved quality of life (QoL), and reduced healthcare expenditures [17].

Porcine placental extracellular matrix (PPECM; InnovaMatrix® AC, Convatec Triad Life Sciences, LLC, Memphis, TN, USA) is a decellularized ECM topical wound covering derived from porcine placental material, which is cleared by the U.S. Food and Drug Administration for wound management. The raw material is sourced from a highly monitored facility that strictly controls for animal age, diet, health, and activity level, which aims to minimize the variability of the source material. During production, PPECM is processed via a proprietary method that balances effective tissue decellularization with the preservation of the native structure of the placental ECM to create a clean and efficient matrix for wound management. Composed of collagen, elastin, laminin, fibronectin, hyaluronic acid, and sulfated glycosaminoglycans, the device functions as a biodegradable, protective collagen wound covering. PPECM is indicated for the management of partial- and full-thickness wounds; pressure ulcers; venous ulcers; diabetic ulcers; chronic vascular ulcers; tunneled/undermined wounds; surgical wounds (donor sites/grafts, post-Mohs surgery, post-laser surgery, podiatric, and wound dehiscence); trauma wounds (abrasions, lacerations, and skin tears); partial-thickness second-degree burns; and draining wounds. The use of PPECM should be discussed with patients for any cultural or religious considerations. PPECM is contraindicated for use in patients with a sensitivity/allergy to porcine materials or collagen, patients with infection in/around the application site, and in third-degree burns.

This study sought to investigate the real-world performance of PPECM via retrospective analysis of relevant electronic health record (EHR) data captured within the US Wound Registry (USWR).

This work was previously presented at the Symposium on Advanced Wound Care (SAWC) Fall 2024, October 2-5, in Las Vegas, Nevada, USA, and at Desert Foot 2024, October 30-November 2, 2024, in Phoenix, Arizona, USA.

## Materials and methods

This real-world study retrospectively analyzed usual clinical care via data obtained from the USWR [11]. The Centers for Medicare and Medicaid Services have recognized the USWR as a qualified clinical data registry since the inception of the program in 2014. As of 2024, over 500 facilities and/or practices in 34 states and Puerto Rico participated in this program via the use of a highly structured EHR purpose-built for wound care (Intellicure, LLC, The Woodlands, TX, USA). Each day, the entire medical record of all patients is electronically transmitted to the USWR, and deidentified research datasets are created from aggregated data.

In this study, data were collected via structured fields in the EHR. The Woodlands Institutional Review Board (The Woodlands, TX, USA) determined that secondary analysis of deidentified data was considered exempt research and informed consent could be waived, pursuant to 45 CFR 46(101)b. The dataset included all hard-to-heal wounds treated at participating clinics from October 10, 2022 through March 25, 2024. This dataset was screened to identify wounds that received at least one application of PPECM. There were no exclusion criteria in this study. There was no mandated follow-up period as data were collected in the usual course of care.

Outcomes and data analysis

The primary outcome of this study was complete wound closure at any time. The EHR platform provides clinicians with structured choices to document wound outcomes (e.g., healed, not healed, transferred care, major amputation, minor amputation, etc.). If a wound outcome was not documented by the clinician, it was imputed by a change in wound size and/or type of tissue exposed. All outcomes were confirmed by central physician adjudication via a review of wound photographs and practitioners’ free-text comments in the EHR. For this study, outcomes were then aggregated into four categories: (a) Closed, defined as having a size reduction of 100% and/or having visible closure on photographic assessment; (b) Major Improvement, defined as wounds with a percent area reduction (PAR) less than 100% and greater than or equal to 85% from the initial documented size; (c) Modest Improvement, Stalled, or Deteriorated (MISD); and (d) Lost-to-Follow-up (LTF) due to insufficient information for outcome determination. Data were also analyzed for the possibility of adverse events, such as patient death, limb amputation, the development of cellulitis or infection, and the reporting of allergic reactions.

Descriptive statistics were calculated for continuous variables, whereas categorical variables were summarized using standard proportional analysis (counts and percentages). Missing wound outcome data were imputed as previously described, where possible, but other missing data were excluded from analysis and reported as unknown. Structured query language was utilized for data extraction and statistical analysis.

## Results

Over the 17-month period analyzed, 76,278 patients with 248,278 wounds were recorded. Of these, 41 patients with 60 wounds received at least one application of PPECM during their wound treatment and were eligible for inclusion (Table 1).

**Table 1:** Patient characteristics (n = 41) Data values are n (%) unless stated otherwise. ^a^ These were the comorbidities officially documented by the physicians using the structured language drop-down menus in the EHRs; any free-text reporting is excluded, and counts may be underreported in this table. EHR, electronic health record

Characteristic	Data value
Patient age, years	
Median (IQR)	74.0 (66.0-80.0)
Patient age	
≥65 years	32 (78.0)
<65 years	9 (22.0)
Sex	
Male	22 (53.7)
Female	19 (46.3)
Method of arrival	
Fully ambulatory	21 (51.2)
Impaired ambulation	18 (43.9)
Bedridden	2 (4.9)
Number of wounds per patient	
Median (IQR)	1.0 (1.0-2.0)
Number of medications taken per patient	
Median (IQR)	6.0 (2.0-9.0)
Most common comorbidities^a^	
Obesity	16 (39.0)
Diabetes	11 (26.8)
Autoimmune disease	5 (12.2)
Hypertension	5 (12.2)
Peripheral arterial disease	5 (12.2)
Nicotine use	5 (12.2)
Depression	1 (2.4)
Hyperlipidemia	1 (2.4)
Illicit drug use	1 (2.4)

Data were contributed by 11 providers at seven sites (five outpatient wound clinics and two skilled nursing facilities) in four states. The characteristics of the 60 study wounds are provided in Table 2, of these 31 (52%) wounds were designated by the treating physician as L/LT. The median wound age at the first PPECM application was 91.0 days (IQR: 61.0-249.0).

**Table 2:** Wound characteristics (n = 60) Data values are n (%) unless stated otherwise. ^a^ Pressure ulcers are recorded in the USWR by location, heels (n = 3), and body (n = 4), and were combined into a single category for this study. ^b^ Defined as a traumatic wound <30 days duration. Traumatic wounds older than 30 days at the time of clinician documentation are classified as chronic ulcers. ^c^ Other wounds included: arterial ulcers (n = 2), diabetic ulcers (not on the foot; n = 2), burns (n = 1), and unspecified other (n = 2). ^d^ As part of the wound assessment process, the EHR prompts the practitioner to respond if the wound is L/LT in a structured drop-down menu for billing purposes. ^e^ Necrotic tissue in the wound bed was not recorded for 12 (20.0) wounds. ^f^ Based on the documentation of green, malodorous, or purulent drainage; erythema; induration; systemic fever; and/or obtaining a wound culture or starting an antibiotic prior to the application of PPECM. CAMP, cellular and/or tissue-based product; DFU, diabetic foot ulcer; EHR, electronic health record; L/LT, limb/life-threatening; PPECM, porcine placental extracellular matrix; USWR, US Wound Registry; VLU, venous leg ulcer

Characteristic	Data value
Wound type	
Chronic ulcer	18 (30.0)
DFU	10 (16.7)
Surgical wound	7 (11.7)
Pressure ulcer^a^	7 (11.7)
VLU	6 (10.0)
Traumatic wound^b^	5 (8.3)
Other wound types^c^	7 (11.7)
Median (IQR) wound size at baseline, cm²	1.50 (0.42-4.69)
Wound age at first PPECM application (n = 54)	
Median (IQR), days	91 (61.0-249.0)
1-180 days	38 (70.4)
180-365 days	7 (13.0)
≥366 days	9 (16.7)
Deepest exposed tissue at the first application of PPECM	
Partial thickness	5 (8.3)
Subcutaneous	49 (81.7)
Muscle	2 (3.3)
Tendon	2 (3.3)
Not reported	2 (3.3)
Wounds documented	
Life/limb-threatening^d^	31 (51.7)
Having 0% of necrotic tissue in the wound bed	2 (3.3)
Having at least one sign of bioburden/infection at or prior to the first PPECM application^f^	52 (86.7)
Wound debridement at or prior to the first PPECM application	41 (68.3)
Management included another CAMP prior to PPECM	11 (18.3)

A review of free-text fields in the EHRs further identified two patients each with a radiation necrosis wound, one patient with two wounds caused by pyoderma gangrenosum, and one patient with four ischemic lower extremity wounds. In addition to the comorbidities documented by treating physicians via he International Classification of Diseases, Tenth Revision, Clinical Modification (ICD-10 CM) diagnosis codes in the EHR (Table 1), a review of free-text fields further revealed that two patients were on dialysis, one patient was on active chemotherapy, one had obvious peripheral ischemia, two were malnourished and bedridden, and one was receiving oxygen and required a soft diet due to swallowing problems.

**Figure 1:**
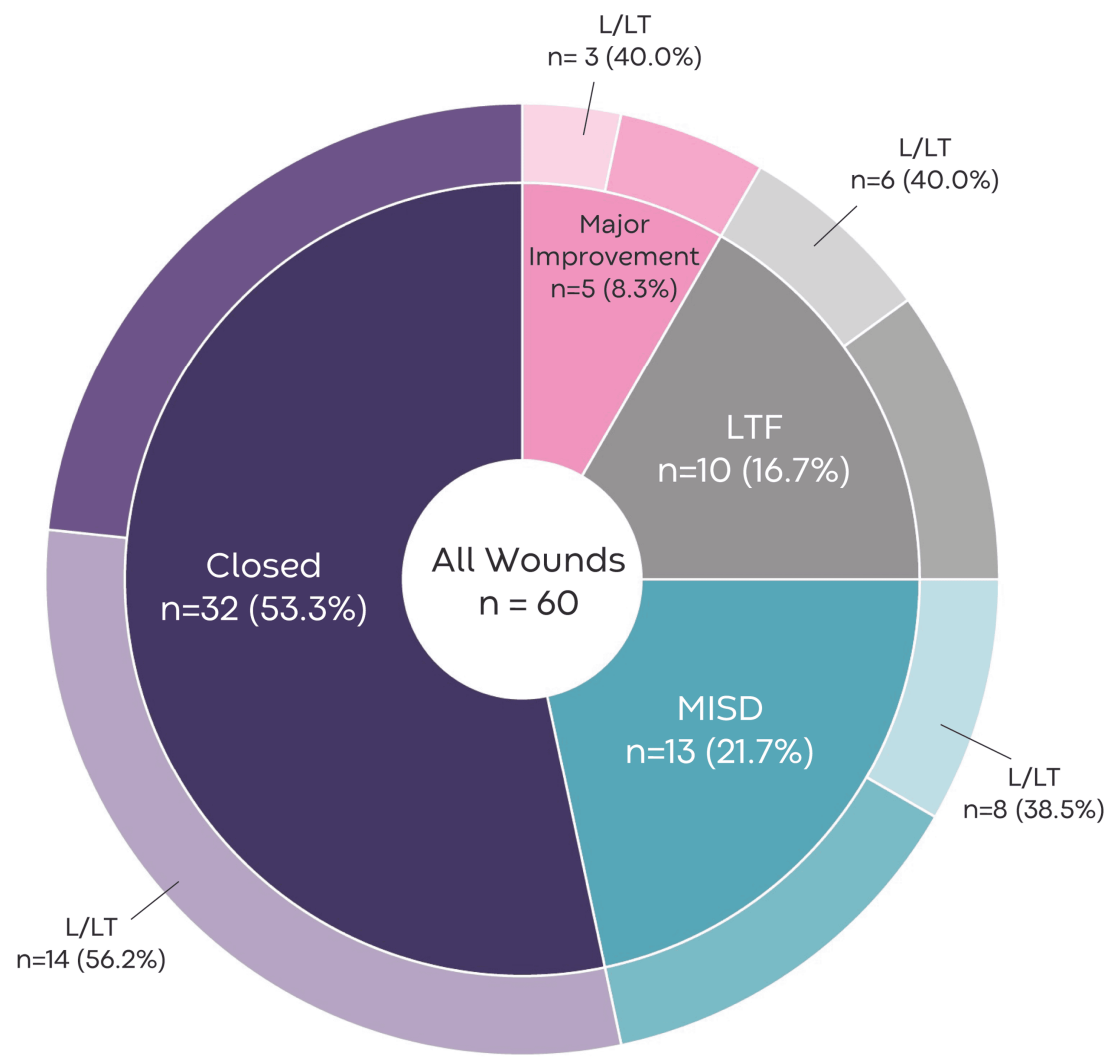
Wound outcomes and proportion that were L/LT L/LT, limb/life-threatening; LTF, Lost to Follow-up; MISD, Modest Improvement, Stalled, or Deteriorated

For the primary outcome of wound closure, remote adjudication determined that 32 (53%) wounds were closed in 24 (58%) patients. A major improvement was observed in five (8%) wounds in four (10%) patients. Thirteen (22%) wounds in nine (22%) patients were classified as MISD, including both pyoderma gangrenosum wounds. Ten (17%) wounds in eight (20%) patients were LTF. Further descriptions of final wound outcomes by L/LT and wound type are provided in Figure 1 and Table 3.

**Table 3:** Outcomes by wound type Data values are n (%) unless stated otherwise. DFU, diabetic foot ulcer; LTF, Lost to Follow-up; MISD, Modest Improvement, Stalled, or Deteriorated; VLU, venous leg ulcer

Wound type	Wounds closed	Major improvement	MISD	LTF
Chronic ulcer (n = 18)	8 (44.4)	2 (11.1)	7 (38.8)	1 (5.5)
DFU (n = 10)	4 (40.0)	1 (10.0)	4 (40.0)	1 (10.0)
Surgical wound (n = 7)	2 (28.6)	0 (0.0)	1 (14.3)	4 (57.1)
VLU (n = 6)	5 (83.3)	1 (16.6)	0 (0.0)	0 (0.0)
Pressure injury (n = 7)	4 (57.1)	0 (0.0)	0 (0.0)	3 (42.9)
Traumatic wound (n = 5)	2 (40.0)	1 (20.0)	1 (20.0)	1 (20.0)
Other (n = 7)	7 (100.0)	0 (0.0)	0 (0.0)	0 (0.0)
Total (n = 60)	32 (53.3)	5 (8.3)	13 (21.7)	10 (16.7)

Eleven wounds had received another CAMP prior to receiving PPECM; of these, closure was observed in five (45%) after management with PPECM, two (18%) showed major improvement, and four (36%) were MISD.

**Figure 2:**
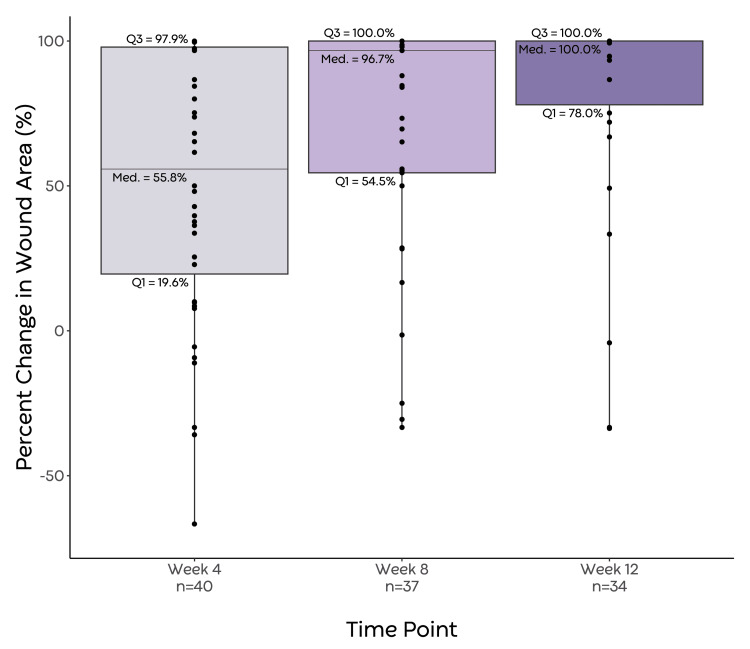
Box-whisker plot depicting PAR at weeks 4, 8, and 12 Increases in wound size over 100% were considered outliers. Individual data points are represented as points on the plot. Med., median; PAR, percent area reduction; Q1, first quartile; Q3, third quartile

Percent wound area reduction was calculated for the cohort at various time points after the initial application of PPECM, using wound measurements that had been recorded as close to the given time point as possible (within ±6 days, maximally) (Figure 2).

Increases in wound area greater than 100% were considered outliers. The median PAR at weeks 4, 8, and 12 was 55.8% (19.6-97.9, n = 40, six outliers), 96.7% (54.5-100, n = 37, three outliers), and 100% (78.0-100, n = 34, three outliers). The median time to wound closure from the first application of PPECM was 53.0 days (IQR: 28.0-105.0). The 60 wounds received a total of 157 PPECM applications, with the median treatment duration occurring over 32.0 days (IQR: 20.0-60.0). The median number of PPECM applications per wound was 2.0 (IQR: 1.0-5.0).

No adverse events or complications occurred.

## Discussion

In this first retrospective clinical study of the real-world use of PPECM, wound closure was observed in 32 (53%) of hard-to-heal wounds. This overall observed closure rate is comparable to rates (57-59%) observed in other real-world studies that evaluated viable placental membranes [14,19]. The majority of wounds identified by this study - 37 (62%) - were not the DFUs, VLUs, and pressure/ulcers typically studied in prospective trials, and therefore this cohort reflects the broad range of wound types typically seen in Medicare beneficiaries [1]. Additionally, since 32 (78%) of patients in our cohort were 65 years of age or older, our data set should be considered highly representative of the Medicare population.

A strength of the current study is that its results were obtained in a medically complex patient population, many of whom had more than one wound, had wounds present for longer than one year, and/or had serious comorbidities, including peripheral arterial disease, dialysis, and autoimmune disease [2]. These characteristics (Table 1) have been shown to impede the wound healing process. In particular, systemic changes associated with diabetes lessen immune response to pathogens, complicating wound healing and increasing the risk of amputation or death [20]. Even if diabetic ulcers heal, the quality of the healed tissue is limited due to peripheral neuropathy and reduced microvascular circulation comorbid to diabetes, and consequently, there is a high risk of re-injury. Furthermore, complications associated with obesity limit neutrophil function, bacterial clearance, and the delivery of antibiotics to the wound [20]. A study by Khalil et al. (2015) on over 3,726 wounds found hypertension prolonged wound healing time [21]. Lifestyle factors such as tobacco, alcohol, and substance abuse also pose a challenge to wound healing. The harmful effects of nicotine on wound healing have been clear for over four decades [22]. Nicotine reduces cutaneous blood flow and oxygen delivery, may accelerate tissue destruction, and increases the risk of infection [23]. Illicit substance and alcohol abuse impair the wound healing process via inhibition of inflammatory and immune responses that underlie the adequate production of Type 1 collagen and also lead to an increase in host protease activity, ultimately leading to a weaker ECM composition in healing wounds [23]. Although these comorbidities are typical of wound care patients encountered in real-world practice, they would be universal grounds for exclusion from relevant RCTs conducted to date.

The outcomes observed in this study (e.g., reduction in wound area, wound closure) were favorable despite the presence of several additional factors known to limit wound healing such as baseline ambulatory status, presence of bioburden, and quality of wound bed preparation. Additionally, no adverse events were observed, suggesting that PPECM is safe. Nearly half of the patients had ambulatory limitations upon arrival, a factor previously shown to be statistically significant for predicting healing failure in most wound types [24]. As of the first PPECM application, the majority of wounds 52 (87%) had documented signs of bioburden, nearly a third (n = 19, 32%) did not have documented debridement, and the presence of necrotic tissue in ≥25% of the wound surface area was documented for 26 wounds (43% of cohort). Only two wounds (3%) were documented as being free of necrotic material in the wound bed at the first application of PPECM. Given that the importance of removing devitalized tissue is well accepted as a cornerstone of high-quality wound care, it would be easy to criticize this as an indication of inadequate wound bed preparation [25]. However, in everyday clinical practice, it may not be possible to achieve a clean wound bed, particularly among ischemic or inflammatory wounds, which may worsen with debridement [26,27]. Despite these limitations, the relatively high rate of wound closure observed in wounds managed with PPECM may be a sign of real-world efficacy.

Complicated wounds are known to require a longer time to heal than captured by the follow-up periods typically used in clinical studies. Previous analysis of USWR RWD and RCT data demonstrated that, at best, only 40% of wounds are expected to heal over a 12-week time frame, if ever [6]. Notably, the favorable healing rates in the current study were obtained only after a median of 53 days. Future research involving PPECM or other novel treatments may benefit from determining whether follow-up periods exceeding 12 weeks can further improve already successful healing rates for hard-to-heal wounds.

Limitations

This real-world analysis was limited by its small sample size and retrospective, noncomparative, uncontrolled design. This analysis utilizes RWD from electronic medical records. Given the variable nature of documentation in real-world clinical practice, the data are at times incomplete with respect to the total cohort (e.g., adjacent and concurrent therapies). In the event that incomplete data was observed in the EHR for a given outcome, the total number of data points available is reported alongside the measured outcome itself. Documentation in the EHR likely underreported the number of comorbid conditions; nevertheless, the RWD collection, analysis, and reporting adhere to recommendations for reporting wound registry data, the United Kingdom’s National Institute for Health and Care Excellence (NICE) framework, and the Strengthening the Reporting of Observational Studies in Epidemiology (STROBE) guidelines for reporting observational data [28,29]. This reporting adherence includes the description of comorbidities, use of other CAMPs, and number of wounds per patient.

Importantly, since the entire EHR was transmitted to the registry, there was no patient selection bias. While all wounds in all patients receiving PPECM were reported, the outcome of nontreated wounds was not included in this analysis. It should be emphasized that, as expected of a real-world study, the approach to care was not mandated by any protocol, nor can it be confirmed that the manufacturer’s application recommendations were followed. As there was no mandated protocol for the SOC, we did not analyze adherence to compression or offloading, which could have affected healing outcomes in the small number of wounds for which these treatments were used [14].

This USWR minimizes bias by collecting research-ready data at point-of-care directly through a purpose-built EHR, which prevents post hoc vetting of outcomes and avoids selection bias, information and recall bias, analytical bias, and channeling bias (confounding by indication) [30].

## Conclusions

This study suggests that in the real world, PPECM may offer clinicians a safe, innovative option for the management of hard-to-heal wounds. More than half of hard-to-heal wounds are closed, despite many of these wounds being L/LT and present in medically complex patients with serious comorbidities. The positive clinical outcomes reported in this first clinical study of PPECM are encouraging for device performance and safety; however, these findings must be confirmed with larger, prospective studies. Further research will help confirm these findings and provide information on other patient-centric outcomes such as QoL, wound pain, and cost-effectiveness. Additional real-world analyses could investigate the use of PPECM in diverse patient populations in the wound care continuum. Prospective randomized trials evaluating the use of PPECM in SOC treatment for DFUs and VLUs are ongoing.
